# Association Between Vitamin D Levels and Hypertensive Disorders of Pregnancy

**DOI:** 10.7759/cureus.97321

**Published:** 2025-11-20

**Authors:** Krishnanand PS, Sangeeta B Singh, Shikhaa Mahajan, Divya Mangla

**Affiliations:** 1 Biochemistry, Amrita Institute of Medical Sciences and Research Center, Faridabad, IND; 2 Biochemistry, Shaheed Hasan Khan Mewati Government Medical College, Nuh, IND; 3 Obstetrics and Gynaecology, Shaheed Hasan Khan Mewati Government Medical College, Nuh, IND

**Keywords:** blood pressure regulation, hypertensive disorders of pregnancy (hdp), maternal health, preeclampsia-eclampsia, vitamin d level

## Abstract

Introduction

Hypertension is a relatively common complication during pregnancy, and preeclampsia represents an important subset of these cases. It is linked to severe complications and adverse outcomes for both mother and baby. Vitamin D, crucial for bone metabolism and immune function, may impact placental function and preeclampsia risk. In Asia, including India, vitamin D deficiency is prevalent among pregnant women, raising questions about its role in hypertensive disorders of pregnancy.

Materials and methods

This cross-sectional study was conducted at Shaheed Hasan Khan Mewati Government Medical College, Nuh, Haryana, India, involving 300 pregnant women divided into hypertensive cases (150) and normotensive controls (150). Cases were further categorized into preeclampsia without severe features, preeclampsia with severe features, and the eclampsia group. Vitamin D levels were measured using the competitive enzyme-linked immunosorbent assay (ELISA) technique. Statistical analysis was performed using IBM SPSS Statistics software, version 26 (IBM Corp., Armonk, NY).

Results

The study found significant differences in mean vitamin D levels among the groups (p = 0.0001). Eclampsia had the lowest mean vitamin D level (8.73 ± 5.10 ng/mL), followed by preeclampsia with severe features (12.54 ± 7.12 ng/mL) and preeclampsia without severe features (13.70 ± 10.86 ng/mL), with controls having the highest level (21.41 ± 5.98 ng/mL). A significant negative correlation (r = -0.6159, p < 0.0001) was observed between the severity of hypertensive disorders and vitamin D levels.

Conclusion

Lower vitamin D levels are significantly associated with increased severity of hypertensive disorders of pregnancy. The findings suggest a potential role for vitamin D in mitigating these complications. Regular monitoring and potential supplementation may improve maternal and fetal outcomes. Further research is needed to confirm these findings and evaluate the effectiveness of vitamin D interventions.

## Introduction

Hypertension is a common complication in 2%-3% of pregnancies. The term "hypertensive disorders of pregnancy" is preferred over "pregnancy-induced hypertension".

Preeclampsia is a condition unique to pregnancy that typically arises after 20 weeks of gestation, marked by elevated blood pressure (exceeding 140/90 mmHg) and the presence of protein in the urine (more than 300 mg over 24 hours). It is a multisystem disorder with unknown causes. Preeclampsia increases the risk of complications such as pulmonary edema, coagulation defects, organ failure, seizures, cerebral hemorrhage, and death. Babies born to mothers with preeclampsia face higher risks of prematurity and being small for gestational age [1].

Around the world, about 4.6% of pregnancies are affected by preeclampsia and 1.4% by eclampsia. In developed countries, preeclampsia happens in about 3.4% of pregnancies, while in developing countries, it can range from 1.8% to 16.7% [2]. The World Health Organization (WHO) reports that the risk of developing preeclampsia is about seven times higher in developing countries than in developed ones. In a secondary analysis focused on low- and middle-income countries, WHO found that preeclampsia rates in India ranged between 2% and 15%. On average, data from individual institutions showed a national rate of around 4.5%. The highest rates were observed in India’s eastern and northeastern regions. In contrast, states such as Karnataka, Himachal Pradesh, Andhra Pradesh, and Haryana reported the lowest incidence of preeclampsia [3].

In normal placental development, cytotrophoblasts invade the myometrium and remodel spiral arteries. In preeclampsia, shallow invasion leads to poor placental perfusion, hypoxia, and endothelial dysfunction, potentially due to abnormal placentation or decidualization. Preeclampsia accounts for 10%-15% of maternal mortality in low- and middle-income countries and about one per 100,000 live births in developed nations. Preeclampsia can be dangerous for both the mother and the baby. For the mother, it may lead to complications such as placental abruption, early delivery, or HELLP syndrome (hemolysis, elevated liver enzymes, and low platelets). For the baby, it can cause problems such as being born too early, low birth weight, or stillbirth [2].

Vitamin D, including D2 (ergocalciferol) and D3 (cholecalciferol), is a group of fat-soluble seco-steroids. Both forms can be obtained from the diet, while D3 is also produced in the skin through sunlight exposure. Vitamin D is converted to its circulating form, calcidiol (25(OH)D), by 25α-hydroxylase, then further hydroxylated in the kidneys by 1α-hydroxylase to form calcitriol (1,25(OH)₂D), the active form of vitamin D. [4]. In Asia, 45%-98% of pregnant women are vitamin D deficient [5]. A recent systematic review on vitamin D deficiency among pregnant women in India reports that the prevalence of vitamin D deficiency ranges from 34 to 96% [6]. Vitamin D plays a key role in bone metabolism, calcium and phosphate absorption, and muscle function. Low serum vitamin D levels during pregnancy are linked to an increased risk of preeclampsia, indicating a potential preventive role for vitamin D supplementation [3].

Vitamin D may influence preeclampsia by regulating genes related to placental function, including implantation, placental invasion, and angiogenesis. It also modulates immune function and inflammation. Maternal vitamin D levels are affected by diet, supplementation, sun exposure, skin pigmentation, and genetics, making vitamin D deficiency a modifiable risk factor for preeclampsia [7].

Vitamin D is distinct among nutrients due to its ability to be synthesized endogenously in the skin upon exposure to ultraviolet B (UVB) radiation, leading to the production of vitamin D3. Humans cannot synthesize vitamin D2. Fish like salmon and mackerel contain D3. Dietary or skin-produced vitamin D is biologically inactive and must be hydroxylated in the liver to 25(OH)D, then further hydroxylated in the kidneys to the active form, 1,25(OH)_2_D, which aids calcium absorption from the intestine [8]. Without the availability of vitamin D, only 10%-15% of dietary calcium and about 60% of phosphorus are absorbed into the blood. Vitamin D sufficiency increases calcium and phosphorus absorption by 30%-40% and 80%, respectively [9].

## Materials and methods

The study was carried out within the Department of Biochemistry at Shaheed Hasan Khan Mewati Government Medical College, Nuh, Haryana, India, in collaboration with the Department of Obstetrics and Gynecology, employing a cross-sectional research design. The study period was from October 2022 to October 2023. The study was conducted after approval from the Institutional Ethics Committee (IEC) (approval number: EC/OA-48/2022), and written informed consent was obtained from all participants. A total of 300 pregnant women were recruited and divided into two groups: 150 hypertensive cases and 150 normotensive controls. The patients were then divided into three categories: preeclampsia without severe features, preeclampsia with severe features, and eclampsia. The sample size was calculated based on a 9% prevalence of preeclampsia, yielding a final minimum sample size of 150, accounting for a 10% nonresponse rate.

Inclusion criteria included women aged 18-45 years with hypertension diagnosed after 20 weeks of gestation. Exclusion criteria were critically ill patients, those over 45 years old, and women with chronic medical conditions or taking vitamin D supplements. Venous blood samples were collected, centrifuged, and analyzed for routine investigations like platelet count, urinary protein, liver function tests, and renal function tests. Vitamin D levels were assessed using a competitive enzyme-linked immunosorbent assay (ELISA) method. The procedure involved incubating serum samples with biotinylated vitamin D and enzyme conjugates, followed by colorimetric detection using a microplate reader.

The free 25(OH)D ELISA utilizes a two-step immunoassay conducted within a microtiter plate. In the initial incubation phase, free 25(OH)D (including both 25(OH)D2 and 25(OH)D3) attaches to the anti-vitamin D antibodies immobilized on the plate’s surface. This process minimally disrupts the natural balance between free and bound 25(OH) vitamin D in the sample.

Following a wash step, a predetermined amount of biotin-labelled 25(OH) vitamin D was introduced into each well. Excess biotinylated 25(OH)D that does not bind was removed by washing, after which a streptavidin-peroxidase conjugate was added. Subsequently, the chromogenic substrate 3,3′,5,5′-tetramethylbenzidine (TMB) was applied.

The reaction was then halted by adding a stop solution, and the absorbance at 450 nm was measured using a spectrophotometer. The level of free vitamin D in the sample was inversely related to the absorbance reading.

Calculation of the results was done using a standard curve created by a microplate reader and plotting the absorbance of reference serum (y-axis) against the corresponding vitamin D concentration (x-axis) in ng/mL. Results were expressed in ng/mL and converted to nmol/L by multiplying by 2.5.

Data were entered in Microsoft Excel and analyzed using IBM SPSS Statistics software, version 26 (IBM Corp., Armonk, NY). The continuous variables were evaluated by mean (standard deviation) or range value when required. The dichotomous variables were presented in number/frequency and were analyzed using the chi-square test. An analysis by the ANOVA test was used to compare the means between two or more groups. Correlation analysis was done using Spearman correlation. A p-value of < 0.05 or 0.001 was regarded as significant.

## Results

In this study, 300 subjects were divided into two groups: 150 cases (patients with hypertensive disorders of pregnancy) and 150 age-matched controls (normotensive pregnant women). The patients were then divided into three categories: 50 with preeclampsia without severe features, 50 with preeclampsia with severe features, and 50 with eclampsia (Table 1).

**Table 1:** Distribution of patients into groups

Patient allocation		
Case	Preeclampsia without severe features	50
	Preeclampsia with severe features	50
	Eclampsia	50
Control		150

The study highlighted significant differences in mean vitamin D levels among the groups (p = 0.0001). Eclampsia cases had the lowest levels (8.73 ± 5.10 ng/mL), followed by preeclampsia with severe features (12.54 ± 7.12 ng/mL) and preeclampsia without severe features (13.70 ± 10.86 ng/mL). The control group had the highest mean vitamin D level (21.41 ± 5.98 ng/mL). Multiple comparison tests confirmed significant differences between each case group and controls (p < 0.0001) (Table 2).

**Table 2:** Comparison of vitamin D levels among case and control groups p<0.05: significant; p>0.05: not significant; *significant p-value

Vitamin D (normal level)	Case			Control	P-value	Chi-square test
	Preeclampsia without severe features	Preeclampsia with severe features	Eclampsia			
20 ng/ml–50 ng/ml	13.70±10.86	12.54±7.12	8.73±5.10	21.41±5.98	P=0.0001*	186.9
Multiple comparison						
Categories		Mean diff.	P-value	95% CI (lower bound)	95% CI (upper bound)	Chi-square test
Preeclampsia without severe features vs. Preeclampsia with severe features		1.16	0.8704	-2.773	5.093	4.53
Preeclampsia without severe features vs. Eclampsia		4.97	0.0068*	1.037	8.903	20.23
Preeclampsia without severe features vs. Control		-7.71	<0.0001*	-11.64	-3.777	99.11
Preeclampsia with severe features vs. Eclampsia		3.81	0.0615	-0.1232	7.743	24.07
Preeclampsia with severe features vs. Control		-8.87	<0.0001*	-12.8	-4.937	96.1
Eclampsia vs. Control		-12.68	<0.0001*	-16.61	-8.747	198.4

A significant negative correlation (Spearman r = -0.6159, p < 0.0001) was found between the severity of hypertensive disorders and vitamin D levels, suggesting that more severe pregnancy complications are associated with lower vitamin D levels (Table 3).

**Table 3:** Correlation between severity of hypertensive disorders of pregnancy and vitamin D levels p<0.05: significant; p>0.05: not significant; *significant p-value

Study	Severity vs. Vitamin D
Spearman's r	-0.6159
95% confidence interval	-0.6834 to -0.5380
P-value	<0.0001*
Chi-square value	198.44

## Discussion

In this study, eclampsia had the highest mean systolic blood pressure (SBP, 174.94±5.42 mmHg) and diastolic blood pressure (DBP, 122.24±2.21 mmHg), followed by severe preeclampsia and preeclampsia without severe features, with the control group showing the lowest values (SBP: 113.27±6.49 mmHg; DBP: 74.68±4.26 mmHg). Statistical analysis revealed significant differences in both SBP and DBP across the groups (p < 0.0001). Aziz et al. reported significantly higher SBP (161±3.2 mmHg) and DBP (106.6±2.19 mmHg) blood pressures in the preeclampsia group compared to controls (120.9±3.3 mmHg and 81.2±1.77 mmHg, respectively) (p<0.0001). [10]. Bongers-Karmaoui et al. and Franchuk et al. observed similar trends, with elevated blood pressure in the preeclampsia and hypertensive groups [11-12]. Uraon and Chandel reported higher SBP (163.10±10.93 mmHg) and DBP (99.67±6.22 mmHg) in the preeclampsia group compared to normotensive controls (110.93±5.45 mmHg and 69.07±4.29 mmHg) with significant differences (p<0.001) [13].

In this study, the eclampsia group had the lowest mean vitamin D level (8.73 ± 5.10 ng/mL), followed by preeclampsia with severe features (12.54 ± 7.12 ng/mL) and preeclampsia without severe features (13.70 ± 10.86 ng/mL), while the control group had the highest level (21.41 ± 5.98 ng/mL), with significant differences among groups (p < 0.0001). Similarly, Sultana et al. found significantly lower mean vitamin D levels in cases (19.13 ± 10.85) compared to controls (41.18 ± 18.61) (p < 0.001) [14]. Nandedkar et al. reported lower vitamin D levels in 95% of cases (mean 31.82 ± 19.82 nmol/L) compared to 72% in controls (mean 52.16 ± 22.18 nmol/L) (p = 0.0001) [15]. Bodnar et al. linked early-pregnancy vitamin D levels <37.5 nmol/L with a 5-fold increase in preeclampsia risk [16]. Dabbaghmanesh et al. found vitamin D levels of 12.7 ± 5 ng/mL in preeclampsia and 15 ± 5.1 ng/mL in controls [17]. Sadin et al. indicated that a vitamin D level <10 ng/mL was associated with a 15-fold increase in preeclampsia risk [18]. Moreover, Abedi et al. found that vitamin D deficiency significantly increased the risk of preeclampsia [19]. Tunc et al. reported similar findings, and Serrano et al. concluded that lower vitamin D levels were associated with a twofold increased risk of preeclampsia [20-21]. This finding is consistent with the results of a meta-analysis, which demonstrated that vitamin D deficiency was linked to a higher risk of developing preeclampsia [22]. Although the protective effect of vitamin D supplementation has not been definitively proven, emerging evidence from studies suggests a potential positive influence of vitamin D intake [23].

Bodnar et al. studied the biological basis of low vitamin D levels and increased risk of preeclampsia [16]. The study found that vitamin D regulates genes involved in placental invasion, implantation, and angiogenesis. Its deficiency may impair immune tolerance, leading to abnormal implantation. Low vitamin D levels are associated with increased inflammation and endothelial dysfunction, contributing to hypertension and proteinuria.

Vitamin D plays a crucial role in managing preeclampsia and eclampsia by potentially reducing their risk and severity. Evidence suggests that adequate vitamin D may improve maternal and fetal outcomes through its effects on blood pressure regulation, inflammation reduction, and immune function. However, more research is needed to establish optimal dosages, timing, and biological mechanisms. Despite these uncertainties, considering vitamin D status in pregnancy management could enhance maternal and neonatal health outcomes.

This study has several limitations that should be considered. First, the sample size of 300 participants may not fully capture the heterogeneity of pregnant populations, which could limit the generalizability of the findings. Additionally, the cross-sectional design restricts the ability to establish causal and temporal relationships between vitamin D levels and hypertensive disorders of pregnancy. The reliance on participants from selected hospitals or clinics may also introduce selection bias, as it may not accurately reflect the broader population of pregnant women. Furthermore, the study may not have comprehensively accounted for potential confounding variables such as dietary intake, sunlight exposure, and other lifestyle factors that can influence vitamin D levels.

## Conclusions

The present study highlights a significant association between lower vitamin D levels and the severity of hypertensive disorders of pregnancy, including preeclampsia and eclampsia. Patients with more severe forms of these disorders exhibited markedly lower vitamin D levels compared to normotensive controls. The data suggest that vitamin D deficiency may be an important factor in the development and progression of these complications. A negative correlation was observed between vitamin D levels and the severity of pregnancy conditions, reinforcing the potential role of vitamin D in mitigating hypertensive disorders. These findings emphasize the need for routine monitoring of vitamin D levels in pregnant women, particularly those at higher risk.
